# Correcting for outcome reporting bias in a meta-analysis: A meta-regression approach

**DOI:** 10.3758/s13428-023-02132-2

**Published:** 2023-07-24

**Authors:** Robbie C. M. van Aert, Jelte M. Wicherts

**Affiliations:** Department of Methodology and Statistics, Tilburg University, P.O. Box 90153, 5000 LE Tilburg, The Netherlands

**Keywords:** Outcome reporting bias, Meta-analysis, Meta-regression, Researcher degrees of freedom

## Abstract

Outcome reporting bias (ORB) refers to the biasing effect caused by researchers selectively reporting outcomes within a study based on their statistical significance. ORB leads to inflated effect size estimates in meta-analysis if only the outcome with the largest effect size is reported due to ORB. We propose a new method (CORB) to correct for ORB that includes an estimate of the variability of the outcomes’ effect size as a moderator in a meta-regression model. An estimate of the variability of the outcomes’ effect size can be computed by assuming a correlation among the outcomes. Results of a Monte-Carlo simulation study showed that the effect size in meta-analyses may be severely overestimated without correcting for ORB. Estimates of CORB are close to the true effect size when overestimation caused by ORB is the largest. Applying the method to a meta-analysis on the effect of playing violent video games on aggression showed that the effect size estimate decreased when correcting for ORB. We recommend to routinely apply methods to correct for ORB in any meta-analysis. We provide annotated R code and functions to help researchers apply the CORB method.

## Introduction

There is ample evidence that findings reported in the psychological literature are biased. For example, the vast majority of published studies in psychology report statistically significant findings (Fanelli, 2010, 2012; Sterling et al., 1995) while the average low statistical power of studies in the psychological literature would imply that most studies should yield non-significant findings (Bakker et al., 2012; Cohen, 1990). Moreover, 100 key findings in psychology were recently replicated to study the replicability of psychological science in the Reproducibility Project: Psychology (Open Science Collaboration, 2015), and effect sizes of the replicated studies were substantially smaller than those of the original studies (correlation coefficient 0.197 vs. 0.403).

The most prominent explanation for the overrepresentation of statistically significant effect sizes in the literatuThere are no conflicts of interest or competingre is the tendency of editors and reviewers to more positively evaluate statistically significant studies (with a *p*-value below the $\alpha$-level) compared to non-significant studies, but researchers also appear less inclined to submit statistically non-significant studies for publication (Cooper et al., 1997; Coursol & Wagner, 1986). The failure to publish studies without a statistically significant effect size, also known as publication bias, is widely understood to create bias in the literature. Additional sources of bias might emerge if researchers are motivated (or feel pressured by a publication system that is still strongly focused on statistical significance) to analyze their data in such a way that it yields a statistically significant effect size. Importantly, multiple analysis approaches are often valid and defensible (Steegen et al., 2016). For instance, 29 analysis teams were asked in a so-called many analyst project to analyze the same data to answer the research question whether referees in football are more likely to give dark skinned players a red card than white skinned players (Silberzahn & Uhlmann, 2015; Silberzahn et al., 2018). The results obtained by the analysis teams varied widely with odds ratio as observed effect size varying from 0.89 to 2.93. Moreover, no analysis approach was deemed to be the best approach and multiple approaches were evaluated as defensible according to the analysis teams who peer reviewed each other’s analysis.

The leeway researchers have to make decisions in the process of setting up a study, analyzing data, and reporting the results are often called *researcher degrees of freedom* or *p-hacking* if this leeway is purposively used to obtain statistical significance (Simmons et al., 2011; Wicherts et al., 2016). John et al., 2012 studied the self-admission rate and defensibility of 10 researcher degrees of freedom related to analyzing data in a sample of 2,000 psychologists employed at universities in the United States. The researcher degree of freedom that was most admitted (63.4%) was “in a paper, failing to report all of a study’s dependent measures” and the vast majority of researchers who admitted it deemed this decision to be defensible (1.84, standard deviation 0.39 on a scale ranging from 0 = not defensible to 2 = defensible). A replication of this study in Italy revealed that the prevalence of admitting not reporting all dependent measures was slightly lower albeit substantial (47.9%, Agnoli et al. 2017).

Selectively reporting dependent measures will bias the literature, especially if only statistically significant measures are reported. Selectively reporting dependent measures is referred to as *outcome reporting bias* or *outcome switching* in medical research. Outcome reporting bias (ORB) is defined as the bias caused by reporting of outcomes/dependent measures that “is driven by the significance and/or direction of the effect size” (Copas et al., 2014). Publication bias is closely related to ORB, but publication bias refers to the suppression of an entire study from being published whereas ORB is the suppression of outcomes being reported in a study. Recent research by Rodgers and Pustejovsky (2021) and Fernández-Castilla et al. (2021) showed that publication bias tests cannot be applied to test for ORB. The effect sizes of the outcomes within a primary study are dependent whereas existing publication bias methods assume independence.

Direct evidence for ORB has especially been obtained in the literature on medical research (e.g., Lancee et al., 2017; Rankin et al., 2017; Wayant et al., 2017). A systematic review (Dwan et al., 2008; Dwan et al., 2013) identified five articles that studied ORB (Chan, Hróbjartsson, et al., 2004a; Chan, Krleža-Jerić et al., 2004b; Elm et al., 2008; Ghersi, 2006; Hahn et al., 2002). All articles studied ORB by comparing the outcomes that were listed in protocols with the outcomes actually being reported in the final publication. The overarching conclusion based on these five studies is that selective reporting of outcomes is prevalent and that statistically significant outcomes are more likely to be reported than non-significant outcomes. For example, Chan, Krleža-Jerić et al., 2004b and Chan, Hróbjartsson, et al., 2004a studied ORB by comparing protocols approved by Danish ethical committees and funded by the Canadian Institutes of Health Research and concluded that 50% and 31% of efficacy and 65% and 59% of harm outcomes were not sufficiently reported in the final publication for being included in a meta-analysis. Moreover, the odds of statistically significant outcomes being reported in the final publication was more than twice as large as those of non-significant outcomes.

Qualitative research revealed that common reasons for not reporting all outcomes are that the results are deemed uninteresting, a too small sample size for a particular outcome, and space limitations by the journal (Smyth et al., 2011). Furthermore, not reporting all outcomes may also be instigated by reviewers and/or editors who request to only report particular outcomes. Some researchers also indicated that they were unaware of the negative consequences of not reporting all outcomes, which is no surprise given the literature on hindsight biases combined with findings highlighting poor statistical intuitions (Bakker et al., 2016; Tversky & Kahneman, 1971).

Research on ORB is more limited in the literature on psychological research, most likely because of the common lack of transparent practices like data sharing and preregistrations (Hardwicke et al., 2022), which would enable meta-scientific studies of ORB. Franco et al., 2016 compared the protocols of 32 psychology experiments with the final publication that ended up in the literature. Fewer outcomes were reported in 72% of the final publications than were listed in the protocol. LeBel et al. (2013) studied ORB by emailing corresponding authors of articles published in prominent psychology journals and asking them whether they had fully disclosed information about the included outcomes as well as data exclusions, sample size, and conditions. Between 20% and 87.2% of the authors indicated to not have reported all the outcomes in their final publication. O’Boyle et al. (2017) compared hypotheses that were tested in dissertations with the corresponding publications. Their results also provide evidence for ORB, because 44.9% of the reported hypotheses in dissertations were statistically significant compared to 65.9% in the publications implying that the results of hypothesis tests were selectively reported.

Multiple methods have been developed to correct for ORB in a meta-analysis (Bowden et al., 2010; Hutton & Williamson, 2000; Jackson et al., 2005). The method developed by Copas and colleagues (Copas et al., 2014, 2019) is the recommended method by the Outcome Reporting Bias in Trials (ORBIT) team. This method requires researchers to first classify all the studies in the meta-analysis according to the risk of bias. For the studies at high risk of bias, it is assumed that outcomes were measured but failed to pass the statistical significance threshold and were not reported. The log likelihood function of either the equal-effect or random-effects model is extended to correct for ORB by incorporating the probability of outcomes in high risk of bias studies being not statistically significant. Drawbacks of this method are that each primary study in a meta-analysis has to be classified according to the risk of bias and it relies on the strong assumption that outcomes were not reported in any study that is at high risk for bias. Another approach is to extend existing publication bias methods to a multivariate meta-analysis model such that they allow for multiple correlated effect sizes within a primary study. Rodgers and Pustejovsky (2021) and Fernández-Castilla et al. (2021) extended existing publication tests by implementing these in a multilevel meta-analysis model or using robust variance estimation.

The goal of this paper is to introduce a new meta-analytic method that can be used as a sensitivity analysis to correct effect size for ORB. The introduced “Correcting for Outcome Reporting Bias” (CORB) method is a meta-regression analysis (Borenstein et al., 2009; Raudenbush, 2009) that includes as a moderator the effect size variability in the outcomes’ effect size of a primary study. The rationale of CORB is that primary studies with a large variability in the outcomes’ effect size are more prone to bias, and that we can correct for this bias by including this variability as a moderator in a meta-regression model. CORB corrects for the bias in effect size estimates caused by ORB by regressing out the effect of researchers choosing to report desirable outcomes among a set of outcomes based on statistical significance or effect size. Meta-analysts are generally familiar with meta-regression analysis, so CORB is easy to understand and straightforward to apply. CORB is different from the method proposed by Copas and colleagues (Copas et al., 2014, 2019), because it does not require classifying the risk of bias of the primary studies. Moreover, CORB models ORB directly rather than extending existing publication bias methods by allowing for multiple correlated effect sizes within a primary study by using a multilevel meta-analysis model or robust variance estimation.

The outline for the remainder of the paper is as follows. We first describe relevant meta-analysis models and introduce our new CORB method to correct for ORB in a meta-analysis. Subsequently, we describe a Monte-Carlo simulation study to examine the statistical properties of the proposed method. We illustrate the method using a meta-analysis on the effect of playing violent video games on aggressive cognition and end with a conclusion and discussion section.

## Meta-analysis models

### Random-effects model

The random-effects model assumes that *k* independent effect size estimates (i.e., $y_i$, *i*=1, ..., *k*) on the same relationship are included in a meta-analysis. The statistical model can be written as (Borenstein et al., 2009; Raudenbush, 2009)

$$\begin{aligned} y_i = \theta _i + \epsilon _i, \end{aligned}$$

where $\theta _i$ consists of the mean $\mu$ of the distribution of true effects and the random effect $\mu _i$ indicating the difference between $\mu$ and the *i*th primary study’s true effect. Furthermore, $\epsilon _i$ is the study specific sampling error. Common assumptions are that $\mu _i \sim N(0,\tau ^2)$ where $\tau ^2$ is the between-study variance in true effect size and $\epsilon _i \sim N(0,\sigma _i^2)$ where $\sigma _i^2$ is the sampling variance of the *i*th primary study’s effect size estimate. This sampling variance is estimated in practice and then assumed to be known. The $\mu _i$ and $\epsilon _i$ are assumed to be mutually independent. Note that the random-effects model simplifies to an equal-effect model (also known as fixed-effect model) if $\tau ^2 = 0$.

### Random-effects meta-regression model

The estimate of $\mu$ is usually the primary interest of researchers. However, the estimate of $\tau ^2$ for assessing whether the primary study’s true effect sizes are different is of equal importance (Higgins et al., 2009). Between-study variance in true effect size can be explained by adding moderator variables to the random-effects model. This random-effects meta-regression model (also known as mixed-effects model) can be written as (Borenstein et al., 2009; Raudenbush, 2009)

1 $$\begin{aligned} y_i = \beta _0 + \beta _1 x_{i1} + ... + \beta _q x_{iq} + \mu _i + \epsilon _i, \end{aligned}$$

where $\beta _0$ is the intercept and $\beta _1$ up to $\beta _q$ are the regression coefficients of moderators $x_{i1}$ up to $x_{iq}$, respectively. The same distributional assumptions as for the random-effects model apply, but $\tau ^2$ is now the *residual* between-study variance in true effect size.

### Multivariate random-effects meta-analysis

The random-effects model and random-effects meta-regression model both assume that the sampling errors (i.e., $\epsilon _i$) are independent. A violation of this assumption occurs when multiple effect sizes are computed based on the same sample. For example, the independence assumption is violated in a meta-analysis on general proficiency if effect sizes for both math and reading proficiency are computed in the primary studies and both are included in the meta-analysis. The multivariate random-effects meta-analysis takes this dependence into account, and its statistical model can be written as (Gleser & Olkin, 2009; Hedges & Olkin, 1985)

$$\begin{aligned} y_{ij} \sim MVN(\theta _{ij}, \textbf{S}_i) \end{aligned}$$

where *MVN* denotes the multivariate normal distribution, $y_{ij}$ is the effect size estimate of the *j*th outcome in the *i*th primary study with *j*=1, ..., *p* outcomes, $\theta _{ij}$ is the true effect size of the *j*th outcome in the *i*th primary study, and $\textbf{S}_i$ is the within-study variance-covariance matrix of the *i*th primary study with the sampling variance of the outcomes’ effect size estimates on the diagonal of $\textbf{S}_i$ and their covariances off-diagonal. Elements of $\mathbf {S_i}$ are again estimated in practice and then assumed to be known.

The $\theta _{ij}$ may be different for each study in the multivariate random-effects model,

$$\begin{aligned} \theta _{ij} \sim MVN(\mu _j, \varvec{\Sigma }) \end{aligned}$$

where $\mu _j$ is the mean of the distribution of true effects of outcome *j* and $\varvec{\Sigma }$ is the between-study variance-covariance matrix with the between-study variance in the outcomes’ true effect sizes on the diagonal of $\varvec{\Sigma }$ and their covariances off-diagonal. The multivariate random-effects meta-analysis model simplifies to a multivariate equal-effect meta-analysis if all elements of $\varvec{\Sigma }$ are equal to zero.

### Multivariate random-effects meta-regression model

Multivariate random-effects meta-analysis can also be extended to allow for the inclusion of moderators in a so-called multivariate random-effects meta-regression model. The primary study’s true effect sizes then become a regression equation analogous to extending the random-effects model to the random-effects meta-regression model. That is, the marginal multivariate random-effects meta-regression model is (e.g., Houwelingen et al., 2002; Jackson et al., 2013)

2 $$\begin{aligned} y_{ij} \sim MVN(\textbf{X}_i \pmb {\beta }, \textbf{S}_i + \varvec{\Sigma }) \end{aligned}$$

where $\textbf{X}_i$ is the design matrix of the *i*th study consisting of *p* rows and $q+1$ columns and $\pmb {\beta }$ is a $q+1$ vector of regression coefficients.

## The new CORB method to correct for outcome reporting bias

### Basic idea

Extreme ORB implies that only the outcome with the largest effect size gets reported and all other outcomes remain unreported. We illustrate the overestimation of true effect size caused by extreme ORB by varying the number of outcomes and the correlations among the outcomes in a simulation. Data were generated assuming a true effect size of $\rho = 0$ and sample size of 40. Correlation coefficients were estimated between one variable and all outcome variables, and the largest correlation coefficient was stored. This procedure was repeated 100,000 times and the average correlation across all repetitions was computed.

**Fig. 1:**
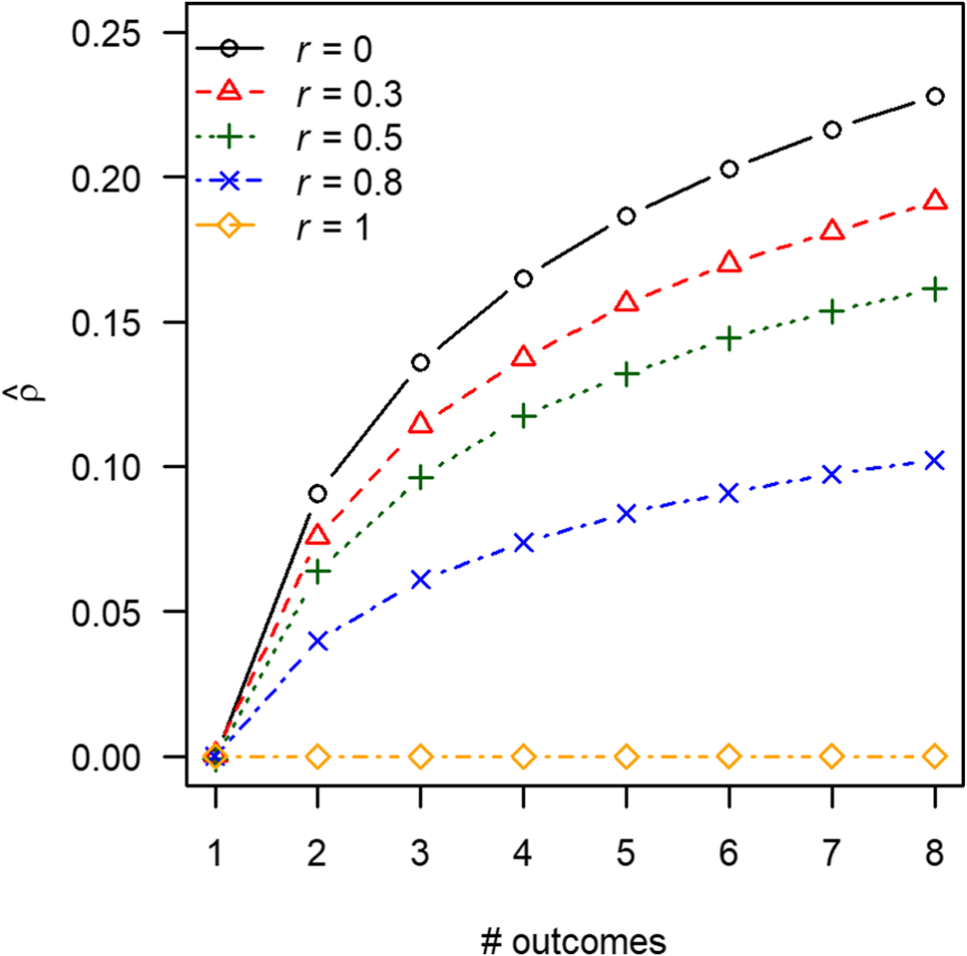
Overestimation of true effect size (Pearson correlation coefficient) caused by outcome reporting bias for varying values of the correlations (*r*) among the outcomes and the number of outcomes. These results were based on a sample size of 40 and true effect size equal to $\rho = 0$. R code of this figure is available at https://osf.io/umnaq/

Figure 1 shows the overestimation caused by extreme ORB as a function of the number of outcomes (*x*-axis) and the correlation among the outcomes (lines in Fig. 1). Figure 1 illustrates that the overestimation caused by ORB is the largest if the outcomes are independent and the number of outcomes is large (maximum overestimation 0.228). The overestimation is zero if the correlation among outcomes is *r* = 1, because all estimated correlation coefficients are then identical. Hence, overestimation decreases as the correlation among the outcomes increases, because the variability in observed effect size estimates decreases as a function of the correlation among the outcomes.

The CORB method uses the variability among outcomes to correct for ORB. That is, we propose to include a measure of the variability of the outcomes’ effect size (e.g., variance or standard deviation of these effect sizes) as a moderator in a meta-regression model. This can be either included in the random-effects meta-regression model in (1) in case all studies report one outcome or the multivariate random-effects meta-regression model in (2) if multiple outcomes are reported in a study. The rationale behind CORB is that the intercept of such a meta-regression analysis is an estimate corrected for ORB, because the intercept is the effect size estimate where the variability of the outcomes equals zero. Hence, the method’s correction for ORB is the largest if the outcomes are independent (i.e., highly variable outcomes’ effect sizes) and the method does not correct for ORB if the outcomes are perfectly correlated (i.e., no variability among outcomes’ effect size).

The rationale behind the method is akin to Egger’s regression test (Egger et al., 1997) and PET-PEESE (Stanley & Doucouliagos, 2014) where a measure of a primary study’s precision is included as a moderator in the random-effects meta-regression model to test and correct for small-study effects. Small-study effects refer to the tendency of small primary studies to be accompanied with large effect sizes, and publication bias is one of the possible causes for these small-study effects (Egger et al., 1997; Sterne et al., 2005). These methods to correct and test for small-study effects, in contrast to CORB, assume that the effect size of a single outcome has been computed and reported for each study. These methods focus on the relationship between the effect size and the variability in the effect size *between* studies whereas CORB focuses on the variability of outcomes *within* studies.

An important assumption of CORB is that the true effect size of the outcomes *within* a primary study are the same. This implies that all $\theta _{ij}$ are assumed to be equal to each other in the *i*th primary study. This assumption of equal true effect size within a primary study is not an unrealistic assumption as multiple scales usually exist to measure a single construct. Multiple test batteries, for example, exist to measure IQ. Moreover, existing scales are frequently modified by, for example, removing items, (e.g., Elson et al., 2014; Flake & Fried, 2020), which also creates multiple outcomes that are likely measuring the same construct. The effect size based on the initial unmodified scale is one outcome and the effect size(s) based on modified versions of the scale are other outcomes. The assumption of equal true effect size within a primary study also aligns with the notion that selecting an outcome a priori is arbitrary so that no prior reasons exist to choose between the outcomes but rather that the researchers choose an outcome based on what is observed in the data. A violation of this assumption biases the effect size estimation of CORB. However, the correction of effect size estimates by CORB is conservative in case of a violation, because the additional variability that is caused by the within-study variance in true effect sizes is not taken into account. Note that the true effect size *between* studies can be either fixed or random in the multivariate meta-analysis model.

### Estimating variability of the outcomes’ effect size

A key element of the proposed method is the estimation of the variability of the outcomes’ effect size. We describe four options for estimating this variability using the observed effect sizes in a primary study, an estimator of the population variance, an estimator of the variance of the difference between two correlated effect sizes, and a bootstrap procedure. Estimating this variability using the latter three options requires information about the correlation among the outcomes. This requirement is not uncommon for meta-analysis methods as the correlation among outcomes is, for instance, also needed in the multivariate meta-analysis model. This correlation is usually not reported in all primary studies (Hedges et al., 2010), so it has to be based on the primary studies that report this correlation or a *guesstimate* (i.e., well-informed guess). A third solution to deal with this unknown correlation is to try different correlations as a sensitivity analysis to assess the impact of varying this correlation.

#### Observed effect size

The most straightforward way to estimate the variability of the outcomes’ effect size is to compute the variance of the reported effect sizes of the outcomes. This is not feasible in cases of extreme ORB where only the effect size of a single outcome is reported. Nevertheless, situations may occur where more than one outcome is reported while at the same time ORB resulted in not reporting all outcomes. An advantage of this approach is that no information is needed about the correlation among the outcomes. Obviously, this option is also feasible when the raw data of the primary studies are available to the meta-analyst.

#### Estimator of the population variance

The second option for estimating the variability is estimating the population within-study variance of the outcomes’ effect size. For each primary study, the outcomes’ effect size is assumed to follow a multivariate normal distribution, so taking the expectation of the variance from a single draw of the multivariate normal distribution provides us with an estimator of the population variance. If we assume an equal true effect size of the outcomes, equal sampling variance of the outcomes, and equal correlations between the outcomes, the estimator of the population variance is equal to (see Appendix A for the full derivation)

$$\begin{aligned} \mathbb {E} \Big [ \frac{1}{p} \sum _{j=1}^p \Big (y_{ij}-\bar{y}_i \Big )^2 \Big ] = \sigma _i^2 (1 - r) \end{aligned}$$

where $\bar{y}_i$ is the mean effect size estimate of the *p* outcomes in the *i*th primary study. This estimator of the population variance is the sampling variance minus the covariance of the multivariate normal distribution and is only a function of the variance of the outcomes’ effect size and the correlation. Hence, the number of outcomes not reported in a primary study is not needed to estimate this variance.

It is important to emphasize that the results of Egger’s regression test (with the sampling variance as moderator) and PEESE are equivalent to CORB with the estimator of the population variance as moderator if all studies contribute a single effect size to the meta-analysis and the correlation between outcomes (i.e., *r*) is assumed to be the same across studies. Egger’s regression test and PEESE then regress the study’s effect size estimate on its sampling variance (i.e., $\sigma ^2_i$) and CORB regresses the effect size estimate on the sampling variance multiplied by a constant.

#### Variance of the difference of outcomes’ effect size

Another measure of the variability of the outcomes’ effect size is the variance of the difference of outcomes’ effect size (hereafter referred to as difference scores). We derive this variance for Fisher-*z* transformed correlations as the effect size measure, because this effect size measure is also used in our Monte-Carlo simulation study and the example where we illustrate CORB. We, however, also provide this variance for raw mean differences in the supplemental materials at https://osf.io/my829/.

The variance of the difference scores for Fisher-*z* transformed correlations is derived by first transforming the observed Pearson correlation coefficient to Fisher-*z* transformed correlations (denoted by *fz*; Fisher (1921)). This transformation is preferred over analyzing Pearson correlation coefficients directly, because it stabilizes the variance and *fz*-correlations approximately follow a normal distribution (Borenstein, 2009; Schulze, 2004). We derive the variance of the difference of two *overlapping* *fz*-correlations That is, we derive the variance of the difference scores between *fz*-correlations of variables *l* and *h* ($fz_{lh}$) and *l* and *m* ($fz_{lm}$). This variance is equal to (see Appendix B for the full derivation)

$$\begin{aligned} Var \Big [fz_{lh} - fz_{lm} \Big ] = \frac{2}{N-3} -2 \frac{r (1-2\rho ^2)-\frac{1}{2} \rho ^2 (1-2 \rho ^2 - r^2)}{(1-\rho ^2)^2 (N-3)} \end{aligned}$$

where *N* is the total sample size, $\rho$ is the population Pearson correlation between variables *l* and *h* and *l* and *m*, and *r* is the estimated Pearson correlation between variables *h* and *m*.

#### Parametric bootstrap

A parametric bootstrap can also be used to estimate the variability of the outcomes’ effect size (Efron & Tibshirani, 1993). That is, we generate outcomes’ effect size under the multivariate random-effects (or equal-effect) meta-analysis model where the within-study variance-covariance matrix $\textbf{S}_i$ is constructed using the observed effect size, sample size, and assumed value for *r* by placing the sampling variance of the observed effect size estimates on the diagonal of $\textbf{S}_i$ and their covariances off-diagonal. Subsequently, we compute the variance of the sampled effect sizes. This procedure is *B* times repeated and the mean of these *B* variances is the estimate of the variance of the outcomes’ effect size.

## Outcome reporting bias index

A meta-regression model with the variability of the outcomes’ effect size estimates as moderator provides an estimate corrected for ORB (intercept) as well as a slope coefficient for the included moderator. This estimated slope coefficient is indicative of the severity of ORB, and it can readily be interpreted in terms of the effect size measure used in the meta-analysis. A large coefficient reflects that there is a strong relationship between the observed effect size estimates and the variability of the outcomes’ effect size. This implies that large effect sizes go along with highly variable outcomes as is expected in case of severe ORB.

We recommend researchers to apply CORB using the effect size measure that is initially used in the meta-analysis. However, transforming the effect sizes can be useful for creating an ORB index. Such an ORB index can be created that allows for comparing the severity of ORB across meta-analyses by first standardizing the observed effect size estimates and moderator such that these have a mean of 0 and variance of 1. Applying CORB based on these standardized variables, provides an ORB index indicating the severity of ORB that is straightforward to interpret. The estimated slope coefficient is analogous to a Pearson correlation coefficient^1^, so large positive scores on the ORB index are indicative of severe ORB. The rules-of-thumb proposed by Cohen (1988) for interpreting the magnitude of a Pearson correlation coefficient can be used for interpreting the ORB index as well.

## Monte-Carlo simulation study

We studied the statistical properties of CORB and compared those to the properties of the (multivariate) random-effects model by means of a Monte-Carlo simulation study. We simulated data with Pearson correlation coefficient and raw mean differences as effect size measures, but we only describe the simulation procedure for generating Pearson correlation coefficients and its results in the paper as correlation coefficients are more common in organizational research. A description of the data generating procedure and the results of the simulations with raw mean differences as the effect size measure are available in the supplemental materials at https://osf.io/my829/. Results of the simulation study using raw mean difference as the effect size measure were comparable to those based on the Pearson correlation coefficients that we report here.

### Method

We simulated data under the multivariate random-effects model where we assumed equal true effect size per outcome within a primary study (i.e., $\theta _{ij} = \theta _i$). For each primary study in the meta-analysis, Pearson correlation coefficients were always computed between variable *l* and one of the *p* outcomes. The true correlation between variable *l* and the *p* outcomes was denoted by $\rho$. Before a true correlation $\rho$ was simulated, it was first transformed to a Fisher-*z* score (i.e., *fz*) using the Fisher-*z* transformation (Fisher, 1921) as meta-analysis models generally assume that the distribution of true effect size in a meta-analysis follows a normal distribution (e.g., Jackson & White, 2018). A primary study’s true effect size $\theta _i$ was then sampled from $N(fz,\tau ^2)$. This true effect size $\theta _i$ was a Fisher-*z* score that was subsequently transformed to a Pearson correlation coefficient using the Fisher-*z* transformation.

We sampled individual participant data from a multivariate normal distribution with mean 0 and variance-covariance matrix that was computed using the generated $\theta _i$, the correlation between the outcomes (*r*), and the variance of the scores in the population. We repeated the procedure above in the rare cases where the variance-covariance matrix was non-positive semi definite caused by a low *r* in combination with a large $\theta _i$ due to large between-study variance in true effect size. The generated data were used to compute Pearson correlation coefficients between variable *l* and the *p* outcomes. Pearson correlation coefficients were also transformed to Fisher-*z* scores to obtain the observed effect size estimates $y_{ij}$. The estimated within-study variance-covariance matrix $\mathbf {S_i}$ was computed with sampling variances equal to those of Fisher-*z* scores (i.e., $1/(N-3)$) and covariances computed using equation (10) in Steiger (1980a). The procedure above was repeated if the estimated within-study variance-covariance matrix $\mathbf {S_i}$ was non-positive semi definite.

Each Pearson correlation coefficient between variable *l* and the *p* outcomes was tested for being different from zero using the *t*-test

$$\begin{aligned} t = r_{ij} \sqrt{\frac{N-2}{1-r_{ij}^2}} \end{aligned}$$

where $r_{ij}$ is the Pearson correlation coefficient between variable *l* and the *j*th outcome in the *i*th primary study. A one-tailed *p*-value was obtained by comparing the observed *t*-statistic with a Student’s *t*-distribution with $N-2$ degrees of freedom. A Pearson correlation coefficient was statistically significant if its *p*-value was lower than $\alpha = .025$. We selected $\alpha = .025$ to resemble common practice of researchers to test a two-tailed hypothesis with $\alpha = .05$ but only report results in the predicted direction.

Two different reporting behaviors by researchers were included in the Monte-Carlo simulation study. The first behavior reflected researchers who always report at least the outcome in a primary study with the lowest *p*-value. The second behavior refers to researchers who always report at least the first outcome that is statistically significant where the order of the outcomes is random. The outcome with the lowest *p*-value is reported if none of the outcomes is statistically significant. This second behavior resembles a researcher who tests outcomes in a random order and stops testing hypotheses if a statistically significant finding has been observed. In case of extreme ORB, the only outcome that was reported in a primary study was either the outcome with the lowest *p*-value or the first statistically significant outcome depending on the researchers’ reporting behavior. Less severe ORB was simulated by also reporting the initially excluded outcomes in a primary study if a random draw from the binomial distribution with probability $1-orb$ was equal to one where *orb* determined the severity of ORB. The above outlined data generating procedure was repeated till *k* primary studies were generated.

The generated effect sizes $y_{ij}$ were combined using a multivariate random-effects meta-analysis where the true effect sizes were assumed to be the same within-study and random between-studies. That is, the multivariate random-effects meta-analysis was fitted using the “rma.mv()” function of the R package “metafor” (version 2.4-0, Viechtbauer, 2010) with as “random” argument “1 | study” where the object “study” indicates from which study an observed effect size comes from. $\textbf{S}_i$ was used as the primary study’s within-study variance-covariance matrix. We included nine different meta-analysis models in the Monte-Carlo simulation study. The first one was the multivariate random-effects meta-analysis model that does not correct for ORB. The other eight meta-analysis models were different types of CORB using the methods introduced earlier for estimating the variability of outcomes’ effect size. Four used the estimated variance as moderator and four used the standard deviation (i.e., square root of the estimated variance) as moderator. Note that the variance and standard deviation of the observed outcomes’ effect sizes are usually not readily available to the meta-analyst, but these models were included to examine the statistical properties of CORB in the ideal situation.

Estimators of the population variance, variance of the difference scores, and bootstrap procedure required a value for *r*. The true value of *r* was used for computing the variability of the outcomes to examine the statistical properties of the models in the ideal situation. However, we also conducted a sensitivity analysis to study whether the models were sensitive to misspecifications of *r* (see results section and supplemental materials at https://osf.io/my829/). The estimator of the variance of the difference scores and bootstrap procedure also required a value for $\rho$. We used the mean of the observed outcomes’ effect size in the *i*th primary study as an estimate for $\rho$. This was the best estimate of $\rho$ based on the available data, because we assumed that the outcomes’ true effect sizes were the same in each *i*th primary study. Moreover, we believe it resembles how researchers would approach this in practice where $\rho$ is also unknown.

We assessed the bias and mean squared error (MSE) in estimation of $\rho$ by the models included in the Monte-Carlo simulation study. The variance of the individual participant data in the population was fixed to 1. Values for the true effect size $\rho$ were 0 and 0.3 to reflect no and a medium effect according to the rules of thumb by Cohen (1988). The number of primary studies in the meta-analysis (*k*) was varied between 10 and 40 where 40 studies is close to the average number of 38.7 primary studies in meta-analyses published in Psychological Bulletin (van Erp et al., 2017), and 10 was included to study the statistical properties of the proposed method in meta-analysis that are representative for medical research (e.g., Gerber et al., 2007). A vector of sample sizes was used (20, 60, 100, 140, 180) that was repeated *k*/5 times to keep the average sample size the same for different values of *k*. The between-study variance $\tau ^2$ was selected in such a way that the $I^2$-statistic (i.e., proportion of total variance that can be attributed to between-study variance in true effect size, Higgins & Thompson, 2002) was equal to 0, 0.25, 0.5, 0.75, and 0.9. The number of outcomes (*dv*) was 2, 4, and 6, and the correlation between these outcomes *r* was 0.3, 0.5, or 0.9.^2^ The severity of ORB was either $orb = 1$ or 0.5 where 1 implied extreme ORB and 0.5 that initially not reported effect sizes have a probability of 0.5 to be included in the meta-analysis. We also studied the statistical properties of the included methods in the absence of ORB (i.e., $orb = 0$) for a selection of the conditions ($\rho$ = 0 and *k* = 10) to study whether the methods distort the meta-analytic results in case of no ORB.

We programmed the Monte-Carlo simulation study in R (version 4.0.1, Team, 2021) and ran 5,000 replications. The total number of samples in the parametric bootstrap was set to 1,000, and the nearest positive definite within-study variance-covariance matrix was computed using the algorithm of Higham (2002) if the matrix was non-positive semi definite in the bootstrap procedure. The R package “metafor” (version 2.4-0, Viechtbauer, 2010) was used for fitting the (multivariate) random-effects meta-analysis model, “MASS” (version 7.3-51.6, Venables & Ripley, 2002) was used for generating data from a multivariate normal distribution, and “parallel” (Team, 2021), “Rcpp” (version 1.0.4.6, Eddelbuettel, 2013), and “RcppArmadillo” (version 0.9.880.1.0, Eddelbuettel & Sanderson, 2014) were used to speed up the computations. The R package “papaja” (Aust & Barth, 2020) was used for writing the paper. R code of the Monte-Carlo simulation study is available at https://osf.io/fykju/.

### Results

We report the results of the Monte-Carlo simulation study of all conditions of *r*, the number of outcomes, the $I^2$-statistic, and $\rho$ in combination with $k = 10$, $orb = 1$, and the reporting behavior where at least the effect size with the lowest *p*-value was reported in this section. We selected these conditions to show that the proposed method can correct for ORB in the most extreme and challenging conditions. We discuss how the results were affected by the other conditions of $\rho$, *orb*, and reporting behavior at the end of this section. All results are available in the supplemental materials at https://osf.io/my829/.

Non-positive semi definite variance-covariance matrices were needed for generating individual participant data and as within-study variance-covariance matrices when applying the multivariate random-effects model and the bootstrap procedure. The maximum proportion of non-positive semi definite variance-covariance matrices for generating individual participant data was 0.067. The maximum proportion of non-positive semi definite within-study variance-covariance matrices was less than 0.001 and 0.105 for applying the multivariate random-effects model and bootstrap procedure, respectively. Moreover, the median proportion of non-positive semi definite variance-covariance matrices used for data generation and applying the multivariate random-effects model equaled 0 and was less than 0.0001 for the bootstrap procedure. Hence, it is unlikely that the non-positive semi definite variance-covariance matrices have distorted the results.

#### Effect size estimation

**Fig. 2:**
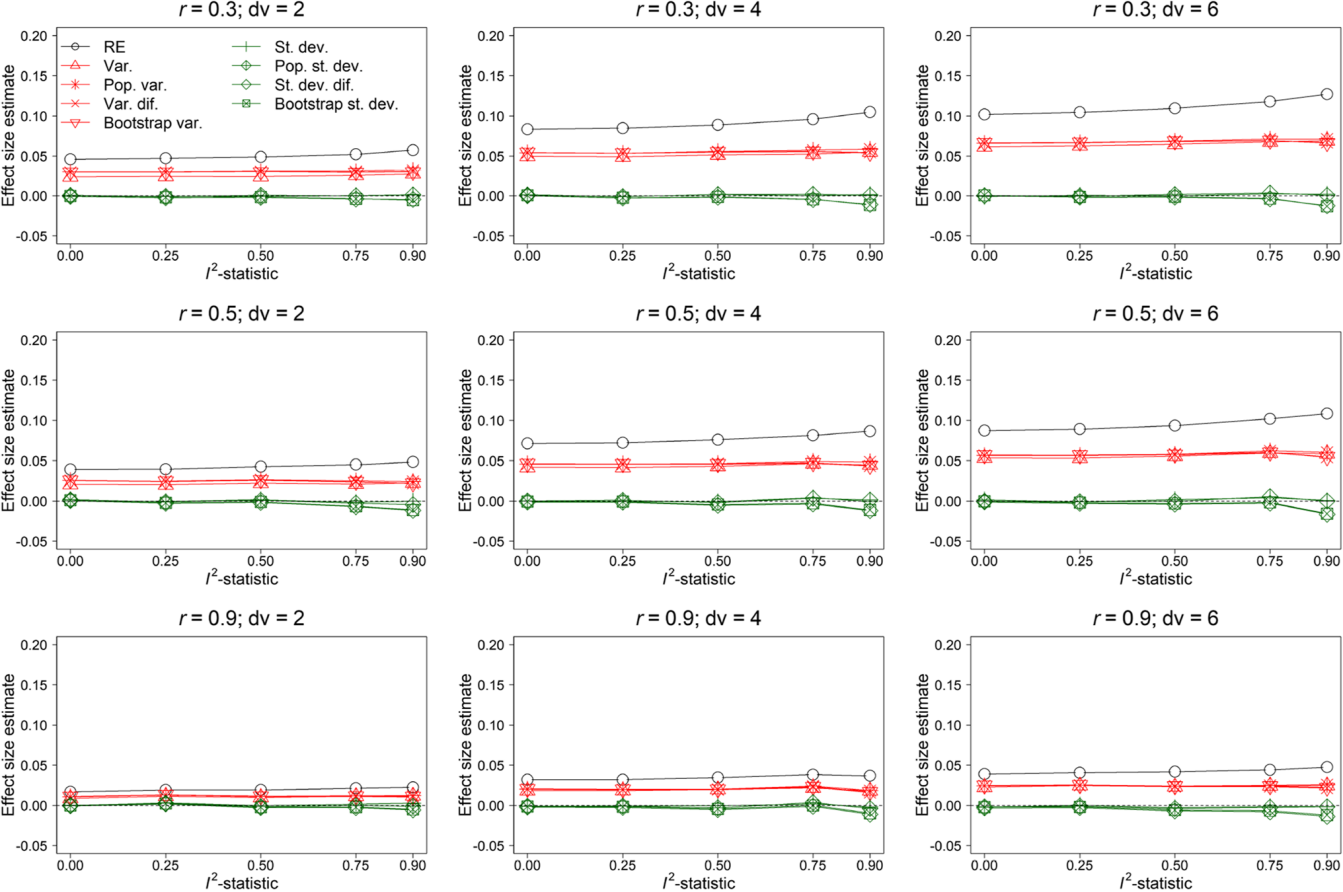
Effect size estimation of the random-effects model (RE) and random-effects meta-regression model with as moderator the variance (Var.) and standard deviation (St. dev.) of the observed outcomes’ effect sizes, an estimate of the population variance (Pop. var.) and standard deviation (Pop. st. dev.), variance of the difference scores (Var. dif.) and standard deviation of the difference scores (St. dev. dif.), and the variance (Bootstrap var.) and standard deviation (Bootstrap st. dev.) obtained with a parametric bootstrap based on 1,000 samples. The results in this figure belong to the condition $\rho = 0$, *orb* = 1, *k* = 10, and reporting behavior where the effect size with the lowest *p*-value was reported

Figure 2 shows the results with respect to estimation of $\rho$ for *k* = 10. The conditions of *r* are varied between the panels in the rows of Fig. 2 and the conditions of *dv* are varied between the panels in the columns. Each plot shows the $I^2$-statistic on the *x*-axis and the average effect size estimate across all replications in the Monte-Carlo simulation study on the *y*-axis. Black lines with open bullets refer to average estimates of the random-effects model. Red lines refer to average estimates of meta-regression models with the variance as moderator and green lines refer to average estimates of models with the standard deviation as moderator. Different estimates of the variability among outcomes’ effect size are indicated by using different symbols. Finally, a horizontal dashed black line denotes the average true effect size.

Bias of the random-effects model (with a maximum bias of 0.127 for the condition $I^2$-statistic = 0.9, *r* = 0.3, and *dv* = 6) was the largest for all conditions included in Fig. 2. Bias of the random-effects model increased as the heterogeneity in true effect size increased (except for condition *r* = 0.9 and *dv* = 4). Models with variance as moderator systematically overestimated the effect size, but this bias was less severe than bias of the random-effects model. Models with standard deviation as moderator yielded accurate effect size estimation. The positive bias of the random-effects model and the models with variance as moderator increased as a function of *dv* and decreased as a function of *r*. This was as expected, because it is most likely to observe a severely overestimated effect size in a study where *dv* is large and *r* is low.

**Fig. 3:**
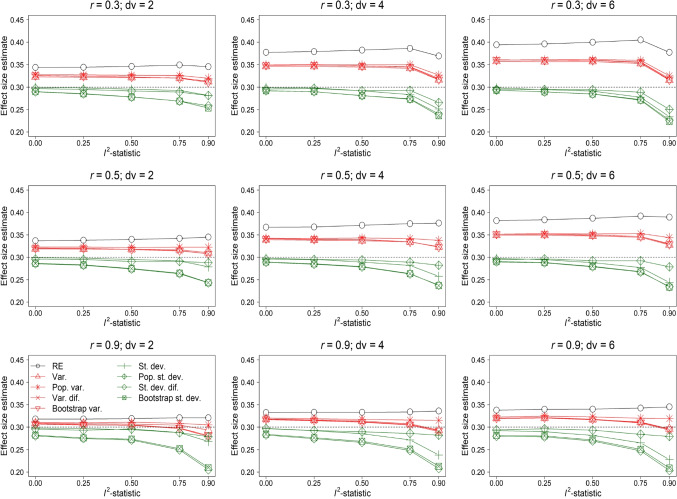
Effect size estimation of the random-effects model (RE) and random-effects meta-regression model with as moderator the variance (Var.) and standard deviation (St. dev.) of the observed outcomes’ effect sizes, an estimate of the population variance (Pop. var.) and standard deviation (Pop. st. dev.), variance of the difference scores (Var. dif.) and standard deviation of the difference scores (St. dev. dif.), and the variance (Bootstrap var.) and standard deviation (Bootstrap st. dev.) obtained with a parametric bootstrap based on 1,000 samples. The results in this figure belong to the condition $\rho = 0.3$, *orb* = 1, *k* = 10, and reporting behavior where the effect size with the lowest *p*-value was reported

**Fig. 4:**
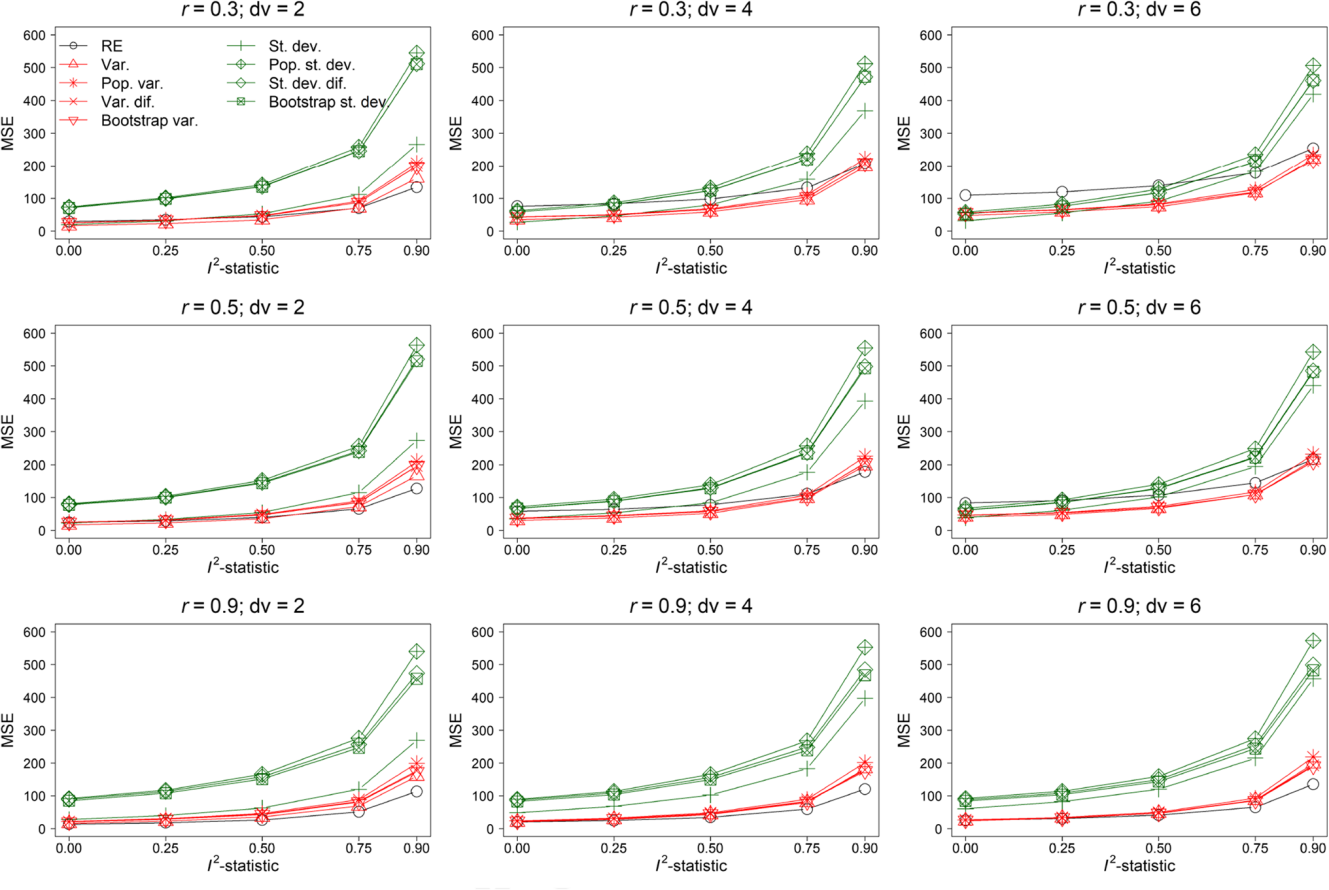
Mean squared error x 10,000 (MSE) of effect size estimation of the random-effects model (RE) and random-effects meta-regression model with as moderator the variance (Var.) and standard deviation (St. dev.) of the observed outcomes’ effect sizes, an estimate of the population variance (Pop. var.) and standard deviation (Pop. st. dev.), variance of the difference scores (Var. dif.) and standard deviation of the difference scores (St. dev. dif.), and the variance (Bootstrap var.) and standard deviation (Bootstrap st. dev.) obtained with a parametric bootstrap based on 1,000 samples. The results in this figure belong to the condition $\rho = 0$, *orb* = 1, *k* = 10, and reporting behavior where the effect size with the lowest *p*-value was reported

The use of the observed variance or standard deviation of the outcomes’ effect size, the population variance estimator, the difference scores, or the bootstrap procedure yielded similar results. However, the models with standard deviation as moderator that were obtained by the bootstrap procedure and difference scores were negatively biased for $I^2$-statistic = 0.9 (maximum negative bias of -0.016 for the condition *r* = 0.5 and *dv* = 6). This was caused by the way the standard deviation was computed in these models. Both the bootstrap procedure and the difference scores require as input $\rho$, which is unknown. We used the mean of the reported correlation coefficients within a primary study as the estimate for $\rho$, but this estimator of $\rho$ was positively biased due to ORB. This resulted in negative bias of the average effect size in these models. The model that used the standard deviation based on the population variance estimator showed less bias in the average effect size, because this estimator of the variance is not a function of $\rho$.

Figure 3 shows the bias in effect size estimation for $\rho = 0.3$. Overestimation was the largest for the random-effects model followed by models with the variance and standard deviation as moderator. However, models with the standard deviation as moderator no longer accurately estimated the effect size. For $I^2$-statistic = 0.9, the estimated effect size was generally less biased with the variance rather than standard deviation as moderator (except for *r* = 0.5 in combination with either *dv* = 4 or 6). Especially models with the standard deviation based on the bootstrap procedure and difference scores substantially underestimated the effect size (maximum bias -0.097 for the difference scores in condition *r* = 0.9 and *dv* = 6).

#### Mean squared error

Figure 4 presents the MSE of effect size estimation for $\rho = 0$ using the same layout as of Figs. 2 and 3. MSE of all methods increased if *dv* and heterogeneity were increased and *r* was decreased. The random-effects model was generally among the methods with the smallest MSE and had the smallest MSE if $I^2$-statistic = 0.9 and *r* = 0.9. This implied that bias in effect size estimation of the random-effects model was compensated by the smaller variance to yield a smaller MSE. There was hardly any difference in MSE between models that used the variance as moderators. These models yielded the lowest MSE for the condition *r* = 0.3 and *dv* = 6. MSE of models with the standard deviation as moderator were the largest and generally comparable. However, MSE of the model using the standard deviation of the observed effect size was smaller than of the other three models with the standard deviation as moderator.

**Fig. 5:**
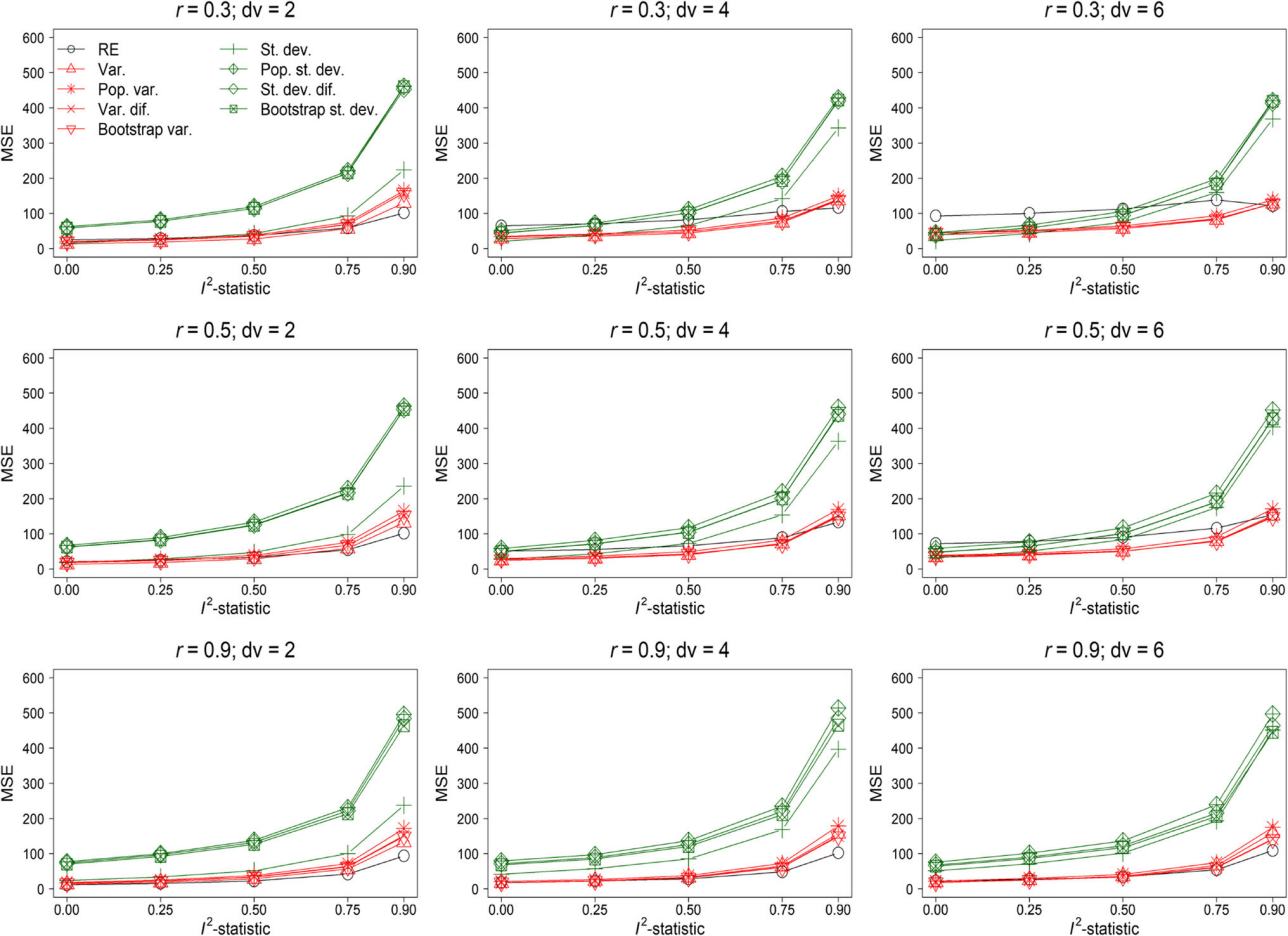
Mean squared error x 10,000 (MSE) of effect size estimation of the random-effects model (RE) and random-effects meta-regression model with as moderator the variance (Var.) and standard deviation (St. dev.) of the observed outcomes’ effect sizes, an estimate of the population variance (Pop. var.) and standard deviation (Pop. st. dev.), variance of the difference scores (Var. dif.) and standard deviation of the difference scores (St. dev. dif.), and the variance (Bootstrap var.) and standard deviation (Bootstrap st. dev.) obtained with a parametric bootstrap based on 1,000 samples. The results in this figure belong to the condition $\rho = 0.3$, *orb* = 1, *k* = 10, and reporting behavior where the effect size with the lowest *p*-value was reported

Figure 5 shows the MSE of effect size estimation for $\rho = 0.3$. MSE of all methods decreased by increasing the true effect size. The patterns in the results were highly similar compared to the results of $\rho = 0$, but the differences between the methods’ MSE were smaller.

#### Other conditions

We summarize the results of the conditions only reported in the supplemental materials (https://osf.io/my829/) in this section. Increasing the number of studies to *k* = 40 hardly affected bias, but showed that MSE of the random-effects model was no longer the lowest except for the conditions with *r* = 0.9 in combination with *dv* = 2 or 4. MSE of the models with variance as moderator were highly comparable and had the lowest MSE in the majority of conditions. These results implied that the method that corrects for ORB benefited more from increasing the number of effect sizes in a meta-analysis than the random-effects model.

Reporting the first statistically significant effect size instead of the one with the lowest *p*-value had only a minor impact on effect size estimation in conditions with $\rho = 0$. This was caused by many of the effect sizes not being statistically significant if $\rho = 0$ and both reporting behaviors ending up with reporting the effect size with the lowest *p*-value. The overestimation of the random-effects model and the models with the variance as moderator decreased if the first statistically significant effect size was reported in combination with $\rho = 0.3$, whereas models with the standard deviation as moderator underestimated the effect size in these conditions. MSE hardly changed across the two reporting behaviors except for the MSE of the random-effects model being comparable to the MSE of models with variance as moderators for the condition where the first statistically significant effect size was reported in combination with *k* = 40, *orb* = 1, and $\rho = 0.3$.

Less severe ORB (*orb* = 0.5) resulted in effect size estimates of all models close to the true effect size if $\rho = 0$. For $\rho = 0.3$, models with the standard deviation as moderator yielded a negative bias especially if $I^2$-statistic = 0.9. The maximum negative bias was -0.121 for the model with the standard deviation based on the difference scores for the condition *r* = 0.9, *dv* = 6, and *k* = 40. MSE of all models was comparable for conditions with *orb* = 0.5 and 1. Bias in effect size estimates of all models was also minimal in case of no ORB (*orb* = 0). MSE of the multivariate random-effects model was now the lowest in all conditions and again the highest for the model with the standard deviation as moderator.

#### Conclusions and recommendations

The Monte-Carlo simulation study showed severe overestimation caused by ORB when the effect size was estimated with the random-effects model. Hence, we recommended to always accompany the results of the (multivariate) random-effects model with a method to correct effect size for ORB if ORB is expected to be present. We generally recommend to use the standard deviation as moderator if the researcher’s main focus is on estimating effect size and to use the variance as moderator if a researcher wants the estimator with the lowest MSE.

A caveat here is that estimates became underestimated if the standard deviation was used as moderator and the true effect size was of medium magnitude ($\rho = 0.3$) in combination with large between-study heterogeneity. Hence, researchers better use the variance as moderator if a medium true effect size in combination with large between-study heterogeneity is expected. In case the magnitude of the true effect size and the extent of between-study heterogeneity is unclear, we recommend to apply and report results of models with both the standard deviation and variance as moderators. Differences between the options for estimating the variability in the outcomes’ effect size were generally small. However, we recommend to estimate the variability using the estimator of the population variance, because it does not require an estimate of $\rho$, can be used for any effect size measure, and is a computationally faster procedure than the bootstrap.

We used the true correlation between the outcomes in our Monte-Carlo simulation study, but this correlation is usually unknown. Hence, we also conducted a sensitivity analysis to assess whether the results of the models were affected by misspecification of the correlation between the outcomes. We reran the simulations for the condition $\rho = 0$, *k* = 10, *dv* = 4, *r* = 0.5, *orb* = 1, and reporting behavior where the study with the lowest *p*-value got reported, but now misspecified the correlation between the outcomes by setting it equal to 0.3 and 0.9. The results of these simulations showed that the models were hardly affected by misspecifying the correlation between the outcomes (see supplemental materials at https://osf.io/my829/ for the results), which is a desirable property of any sensitivity analysis.

## Example

We illustrate CORB by applying it to a meta-analysis on the effect of playing violent video games on aggression. ORB is expected to be a concern in this research field as there is a high degree of flexibility in the measurement of aggression outcomes (Elson et al., 2014; Hilgard et al., 2019; e.g., Przybylski & Weinstein, 2019). The meta-analysis by Anderson et al. 2010) studied the effect of playing violent video games on aggressive affect, aggressive behavior, aggressive cognition, and physiological arousal. We apply CORB to the 40 experiments^3^ included in the meta-analysis with aggressive cognition as outcome. The effect size measure of these primary studies are Pearson correlation coefficients.

CORB was applied to these data using the variance and standard deviation obtained with the estimator of the population variance, the difference scores, and the bootstrap procedure. Only one outcome variable was reported per study, so we could not use the variance or standard deviation of the observed effect size as moderator in CORB. The “puniform” package (van Aert, 2022) contains easy-to-use functions to estimate the population variance, the variance of the difference scores, and the variance using the bootstrap procedure. The “var_pop()” function can be used to estimate the population variance for any effect size measure. Dedicated functions were developed for estimating the variance of the difference scores (“var_dif_fis()” and “var_dif_rmd()”) and applying the bootstrap procedure (“var_boot_fis()” and “var_boot_rmd()”) for Fisher-*z* correlation and raw mean difference as effect size measure.

The “var_pop()” function requires as input the sampling variance of a primary study’s effect size estimate as well as *r*. The function “var_dif_fis()” requires as input the sample size, *r*, and $\rho$ and “var_boot_fis()” requires as input the observed Pearson correlation coefficient of the primary study, *N*, and *r*. Information on the sampling variance, sample size, and observed Pearson correlation coefficient is available in this meta-analysis. We used three different levels for *r* (0.5, 0.7, and 0.9) as a sensitivity analysis and the observed Pearson correlation coefficient for $\rho$ to match the procedure in the Monte-Carlo simulation study. R code of this analysis is available at https://osf.io/rpqus/.

Table 1 shows the results of applying CORB and the random-effects model to the meta-analysis on the effect of playing violent video games on aggressive cognition. Only the results of *r* = 0.7 are presented in Table 1, because the results of *r* = 0.5 and 0.9 were highly similar and therefore only included in the supplemental materials at https://osf.io/my829/. The average effect size estimate of the random-effects model that does not correct for ORB was 0.200 and significantly different from zero (*z* = 10.431, *p* < .001). Applying CORB with the variance as moderator yielded average effect size estimates between 0.153 and 0.158 that were all statistically significant. Using the standard deviation as moderator in CORB resulted in average effect size estimates between 0.091 and 0.114 that were not statistically significant or only marginally significant. The ORB index of all models (last column of Table 1) was close to 0.5 indicating that there was a large correlation between the observed effect size and variability of the outcome’s effect size. To conclude, correcting for ORB using CORB showed that the average effect size became closer to zero but was still at least a small effect according to the rules of thumb by Cohen (1988).

**Table 1:** Results of applying the random-effects (RE) meta-analysis model and CORB to the meta-analysis on the effect of violent video games on aggressive cognition. Results of the variance and standard deviation (St. dev.) based on the estimator of the population variance, difference scores, and bootstrapping are presented

		Estimate	(95% CI)	Test of no effect	ORB index
RE		0.200	(0.163;0.236)	*z*=10.431; $p<0.001$	-
Pop. estimator	Variance	0.158	(0.101;0.214)	*z*=5.386; $p<0.001$	0.505
	St. dev.	0.114	(0.001;0.224)	*z*=1.972; *p*=0.049	0.466
Dif. scores	Variance	0.153	(0.100;0.204)	*z*=5.675; $p<0.001$	0.572
	St. dev.	0.091	(-0.014;0.195)	*z*=1.694; *p*=0.09	0.547
Bootstrap	Variance	0.155	(0.103;0.206)	*z*=5.788; $p<0.001$	0.563
	St. dev.	0.095	(-0.010;0.198)	*z*=1.777; *p*=0.075	0.541

Note: Correlation between outcomes was assumed to be equal to $r=0.7$; between-study variance in the random-effects meta-analysis model was estimated using restricted maximum likelihood estimation; CI = confidence interval; two-tailed *p*-values are reported

## Conclusion and discussion

ORB is a pervasive problem in psychological research (Franco et al., 2016; John et al., 2012) that yields overestimated effect size estimates in primary studies. Combining these primary studies in a meta-analysis results in an overestimated meta-analytic effect size estimate as well. The meta-analytic reporting standards (MARS, Appelbaum et al. 2018) of the American Psychological Association and the Preferred Reporting Items for Systematic Reviews and Meta-Analyses (PRISMA, Moher et al., 2009) both advise researchers to address the risk of biases such as ORB in any meta-analysis. However, the risk of bias is not routinely examined in meta-analyses published in psychological research (Hohn et al., 2019).

The lack of attention for assessing the risk of bias in general and ORB in particular within psychology is likely caused by current methods being difficult to apply. For instance, the method that is nowadays recommended by the ORBIT team to correct for ORB requires researchers to classify the risk of bias in each primary study. This is currently not frequently done in meta-analyses published within psychology. We proposed the CORB method that is easier to apply as it does not require such a classification. The method can be used as a sensitivity analysis to estimate the average effect size by including the variability of the outcomes’ effect size as a moderator in a (multivariate) random-effects meta-regression model. A major advantage of the method is that it is intuitive and easy to apply, because most researchers are familiar with meta-regression.

Results of a Monte-Carlo simulation study revealed severe overestimation of the random-effects meta-analysis if ORB was severe. Hence, we recommend to always accompany the results of the random-effects or equal-effect model with a method to correct for ORB if ORB is expected to be present. Effect size estimation of CORB with the standard deviation as moderator was accurate in the conditions where overestimation caused by ORB was the largest (true effect equal to zero, small correlation among the outcomes, large number of outcomes, and only the outcome with the largest effect size was reported). However, CORB with the standard deviation as moderator generally had larger MSE especially if the number of studies in a meta-analysis was small. In those cases, we found underestimation of effect size if the true effect size was of medium magnitude. Hence, we recommend researchers expecting a medium true effect size to use the variance as moderator in CORB if an estimator is desired with less uncertainty (i.e., smaller MSE), because it had a smaller MSE in the simulations. If a researcher is completely unsure about the magnitude of the true effect, we recommend to apply and report the results of the method with both the standard deviation and variance as moderator. In case a researcher is also interested in other moderating variables, we recommend to include the moderator quantifying the variability of the outcomes’ effect size together with the other moderators. This allows for interpreting the effect of a moderator while controlling for the other included moderators.

A limitation of the proposed method is that the correlation is needed between the outcomes in the primary studies. This information is often not readily available (Hedges et al., 2010), so we usually have to rely on a *guesstimate* of this correlation coefficient or use different values for this correlation coefficient in a sensitivity analysis. It is important to emphasize that misspecifying this correlation coefficient will bias the results of CORB. However, a small Monte-Carlo simulation to study the robustness of CORB to misspecification of the correlation between the outcomes showed that the method’s results were hardly affected by misspecifications. This is in line with previous research that showed that the meta-analytic estimate of a multivariate meta-analysis is not very sensitive to misspecifying the correlation between outcomes in case of complete data (Ishak et al., 2008; Riley, 2009). However, the robustness of CORB to misspecifying the correlation between the outcomes also needs to be studied for other effect size measures than Pearson correlation coefficients such as raw mean differences.

Future research may focus on extending CORB such that the strong assumptions of homogeneous true effect size of the outcomes within a primary study and the same correlation between outcomes can be relaxed. This will increase the flexibility of CORB and makes it more widely applicable. Another opportunity for future research is that CORB opens doors for simultaneously correcting for publication bias and ORB. This is of utmost importance as publication bias methods do not perform well if researcher degrees of freedom are present in the primary studies (Carter et al., 2019; van Aert et al., 2016). Including the variability of outcomes’ effect size as a moderator in a publication bias method such as a selection model approach (Hedges & Vevea, 2005; van Aert & van Assen, 2022) or PET-PEESE (Stanley & Doucouliagos, 2014) is a promising approach to simultaneously correct for publication bias and ORB in a meta-analysis.

Another interesting avenue for future research is that the developed method can serve as a starting point for a general framework to correct for the biasing effects of researcher degrees of freedom in primary studies. The developed method can, in theory, correct for all researcher degrees of freedom that cause overestimation of effect size as long as variability in the outcomes’ effect size can be estimated. That is, the bias of many researcher degrees of freedom is caused by variability in the outcomes’ effect sizes in combination with selective reporting of the outcomes. For example, failing to report all of a primary study’s conditions is another frequently used researcher degree of freedom according to John et al. (2012) and Agnoli et al. 2017). The conditions in an experiment are then selectively reported rather than the outcomes. This potentially also yields overestimated effect size if, for instance, the experimental condition is compared to multiple control conditions and only the control condition resulting in the largest mean difference with the experimental condition gets reported. The developed method can also correct for this overestimation by including the variability of the outcomes’ effect size that is caused by selectively reporting conditions as a moderator in a meta-regression model. Another example is when researchers are running the analyses on multiple subsets of the sample and selectively report the results of a single subset. This also causes additional variability in the outcome’s effect sizes that can be taken into account.

To conclude, we have proposed the easy to apply CORB method to correct the effect size for ORB in a meta-analysis. CORB was able to correct for bias in conditions where overestimation caused by ORB was the largest. To facilitate its use, we included R functions required to apply CORB in the “puniform” package. We hope that the development of CORB draws attention to the prevalent problem of ORB and that it will be routinely used to correct for ORB in meta-analyses.

## Data Availability

R codes of the Monte-Carlo simulation study are available on the Open Science Framework (https://osf.io/wzmh9/). Data of the of the example was borrowed from the meta-analysis by Hilgard et al. (2019), and are available on their GitHub page: https://github.com/Joe-Hilgard/Anderson-meta/blob/master/cleaned_data.txt

## Appendix A

We derive the estimator of the population variance of a single draw from a multivariate normal distribution in this appendix. The expectation of the population variance is equal to

3 $$\begin{aligned} \mathbb {E} \Big [ \frac{1}{p-1} \sum _{j=1}^p \Big ( y_{ij}-\bar{y}_i \Big )^2 \Big ] = \frac{1}{p-1} \Big ( \mathbb {E} \Big [ \sum _{j=1}^p y_{ij}^2 \Big ] - \frac{1}{p} \mathbb {E} \Big [ \Big (\sum _{j=1}^p y_{ij} \Big )^2 \Big ] \Big ). \end{aligned}$$

The expectation of the first term on the right hand side of (3) equals

4 $$\begin{aligned} \mathbb {E} \Big [ \sum _{j=1}^p y_{ij}^2 \Big ] = \sum _{j=1}^p \Big (\theta _{ij}^2+\sigma ^2_{ij} \Big ) \end{aligned}$$

where $\sigma ^2_{ij}$ is the *j*th diagonal element of the within-study variance-covariance matrix $\mathbf {S_i}$, which is the true variance of the *j*th outcome’s effect size. The expectation of the second term on the right hand side equals

5 $$\begin{aligned} \mathbb {E} \Big [ \Big ( \sum _{j=1}^p y_{ij} \Big ) ^2 \Big ] = \mathbb {E} \Big [ \sum _{j=1}^p \sum _{q=1}^p y_{ij} y_{iq} \Big ] = \Big ( \sum _{j=1}^p \theta _{ij} \Big )^2 + \sum _{j=1}^p \sum _{q=1}^p \sigma _{ijq} \end{aligned}$$

where $\sigma _{ijq}$ refers to the element in the *j*th row and *q*th column of the within-study variance-covariance matrix $\mathbf {S_i}$ of the *i*th primary study, which is the covariance between the effect size of outcome *j* and *q*. Hence, the last term of (5) is the sum of all elements of $\mathbf {S_i}$.

Substituting equations (4) and (5) into (3) yields

$$\begin{aligned} \mathbb {E} \Big [ \frac{1}{p-1} \sum _{j=1}^p \Big (y_{ij}-\bar{y}_i \Big )^2 \Big ] = \frac{1}{p-1} \Big [ \sum _{j=1}^p \theta ^2_{ij} + \sum _{j=1}^p \sigma ^2_{ij} - \frac{1}{p} \Big (\sum _{j=1}^p \theta _{ij} \Big )^2 - \frac{1}{p} \sum _{j=1}^p \sum _{q=1}^p \sigma _{ijq} \Big ]. \end{aligned}$$

Upon assuming equal true effect size of the outcomes (i.e., $\theta _{ij} = \theta _i$), equal sampling variance of the outcomes (i.e., $\sigma _{ij}^2 = \sigma _i^2$), and equal covariances between the outcomes (i.e., $\sigma _{ijq} = r \sqrt{\sigma _i^4}$), this simplifies to

$$\begin{aligned} \mathbb {E} \Big [\frac{1}{p-1} \sum _{j=1}^p \Big (y_{ij}-\bar{y}_i \Big )^2 \Big ]= & {} \frac{1}{p-1} \Big [p \theta _i^2 + p \sigma _i^2 - \frac{1}{p} \Big (p \theta _i \Big )^{2} - \frac{1}{p} \sum _{j=1}^p \sum _{q=1}^p \sigma _{ijq} \Big ]\\= & {} \frac{1}{p-1} \Big [p \sigma _i^2 - \frac{1}{p} \Big ( p \sigma _{i}^{2} + (p^2-p) r \sqrt{\sigma _i^4} \Big ) \Big ]\\= & {} \frac{1}{p-1} \Big [ (p-1) \sigma _{i}^{2} - (p-1) r \sqrt{\sigma _i^4} \Big ]\\= & {} \sigma _i^2 (1 - r). \end{aligned}$$

## Appendix B

We derive the variance of the difference between two Fisher-*z* transformed correlation coefficients in this appendix. The two Fisher-*z* transformed correlations are overlapping such that one correlation is based on variables *l* and *h* and the other correlation on variables *l* and *m*. This variance is equal to

$$\begin{aligned} Var \Big [fz_{lh} - fz_{lm} \Big ] = Var \Big [fz_{lh} \Big ] + Var \Big [fz_{lm} \Big ] - 2Cov \Big [ fz_{lh},fz_{lm} \Big ] \end{aligned}$$

where $fz_{lh}$ and $fz_{lm}$ refer to the Fisher-*z* transformed correlation coefficients of variables *l* and *h* and *l* and *m*, respectively.

The first two terms of the equation above, $Var \Big [fz_{lh} \Big ]$ and $Var \Big [fz_{lm} \Big ]$, are equal to $1/(N-3)$, because we assume that both Fisher-*z* transformed correlation coefficients are based on the same sample and that the variables *l*, *h*, and *m* do not contain missing data. The third term, $Cov \Big [ fz_{lh},fz_{lm} \Big ]$, has been derived before (e.g., Dunn & Clark, 1969; Steiger, 1980a, b). Let $\sigma _{lh,lm}$ be the covariance of two overlapping Fisher-*z* transformed correlations (see equation (10) in Steiger (1980a) with slightly adapted notation to avoid a notational conflict)

$$\begin{aligned} \sigma _{lh,lm} = \frac{\Psi _{lh,lm}}{(1-\rho ^2_{lh}) (1-\rho ^2_{lm}) (N-3)} \end{aligned}$$

where $\rho _{lh}$ and $\rho _{lm}$ are the true Pearson correlations between variables *l* and *h* and *l* and *m*, and $\Psi _{lh,lm}$ is *N* times the covariance of the Pearson correlations between variables *l* and *h* and *l* and *m*. $\Psi _{lh,lm}$ is computed with (see equation (3) in Steiger (1980a))

$$\begin{aligned} \Psi _{lh,lm} = \rho _{hm} (1 - \rho _{lh}^2 - \rho _{lm}^2) - \frac{1}{2} (\rho _{lh} \rho _{lm}) (1- \rho _{lh}^2 - \rho _{lm}^2 - \rho _{hm}^2). \end{aligned}$$

Under the assumptions that $\rho _{lh} = \rho _{lm} = \rho$ and $\rho _{hm} = r$, $Var \Big [fz_{lh} - fz_{lm} \Big ]$ is obtained by combining the terms above

$$\begin{aligned} Var \Big [fz_{lh} - fz_{lm} \Big ]= & {} \frac{1}{N-3} + \frac{1}{N-3} - 2\frac{r (1 - \rho ^2 - \rho ^2) - \frac{1}{2} (\rho \rho ) (1- \rho ^2 - \rho ^2 - r^2)}{(1-\rho ^2) (1-\rho ^2) (N-3)} \\= & {} \frac{2}{N-3} -2 \frac{r (1-2\rho ^2)-\frac{1}{2} \rho ^2 (1-2 \rho ^2 - r^2)}{(1-\rho ^2)^2 (N-3)}. \end{aligned}$$

## Footnotes

1 The estimated coefficient is not exactly equal to a Pearson correlation coefficient, because a meta-analysis generally weighs effect sizes by the inverse of the variances. The ORB index is exactly equal to a correlation coefficient if the same weights are used for each effect size in a univariate meta-analysis.

2 We initially also included the condition $r = 0.1$, but it turned out that this often resulted in a non-positive semi definite variance-covariance matrix when generating the individual participant data. This occurred especially in combination with $\rho = 0.3$ and large between-study variance in true effect size. The primary study’s true effect size $\theta _i$ can then be large due to the large between-study variance and the fact that observing a large $\theta _i$ in combination with $r = 0.1$ is unlikely. The problem of a non-positive semi definite variance-covariance matrix was less apparent for larger values of *r*. We reported how often non-positive semi definite within-study variance-covariance matrices were obtained in the results section.

3 Anderson et al. 2010 report in their Table 5 that the full sample meta-analysis with aggressive cognition as outcome consists of 48 experiments. We used the cleaned data by Hilgard et al. (2019) for our analyses who aggregated effect sizes within a study, and therefore meta-analyze 40 rather than 48 experiments.

## References

[CR1] Agnoli F, Wicherts JM, Veldkamp CLS, Albiero P, Cubelli R (2017). Questionable research practices among italian research psychologists. PLOS ONE.

[CR2] Anderson CA, Shibuya A, Ihori N, Swing EL, Bushman BJ, Sakamoto A, Saleem M (2010). Violent video game effects on aggression, empathy, and prosocial behavior in eastern and western countries: A meta-analytic review. Psychological Bulletin.

[CR3] Appelbaum M, Cooper H, Kline RB, Mayo-Wilson E, Nezu AM, Rao SM (2018). Journal article reporting standards for quantitative research in psychology: The APA publications and communications board task force report. The American Psychologist.

[CR4] Aust, F., & Barth, M. (2020). Papaja: Prepare reproducible APA journal articles with R Markdown. (0.1.0.9942 ed.). Retrieved from https://github.com/crsh/papaja

[CR5] Bakker M, Hartgerink CHJ, Wicherts JM, van der Maas HLJ (2016). Researchers’ intuitions about power in psychological research. Psychological Science.

[CR6] Bakker M, van Dijk A, Wicherts JM (2012). The rules of the game called psychological science. Perspectives on Psychological Science.

[CR7] Borenstein M, Cooper H, Hedges LV, Valentine JC (2009). Effect sizes for continuous data. The Handbook of Research Synthesis and Meta-Analysis.

[CR8] Borenstein, M., Hedges, L. V., Higgins, J. P. T., & Rothstein, H. R. (2009). Introduction to meta-analysis. John Wiley & Sons, Ltd.

[CR9] Bowden J, Jackson D, Thompson SG (2010). Modelling multiple sources of dissemination bias in meta-analysis. Statistics in Medicine.

[CR10] Carter EC, Schönbrodt FD, Gervais WM, Hilgard J (2019). Correcting for bias in psychology: A comparison of meta-analytic methods. Advances in Methods and Practices in Psychological Science.

[CR11] Chan A-W, Hróbjartsson A, Haahr MT, Gøtzsche PC, Altman DG (2004). Empirical evidence for selective reporting of outcomes in randomized trials: Comparison of protocols to published articles. JAMA.

[CR12] Chan A-W, Krleža-Jerić K, Schmid I, Altman DG (2004). Outcome reporting bias in randomized trials funded by the canadian institutes of health research. Canadian Medical Association Journal.

[CR13] Cohen, J. (1988). Statistical power analysis for the behavioral sciences (2nd ed.). Lawrence Erlbaum Associates.

[CR14] Cohen J (1990). Things i have learned (so far). American Psychologist.

[CR15] Cooper H, DeNeve K, Charlton K (1997). Finding the missing science: The fate of studies submitted for review by a human subjects committee. Psychological Methods.

[CR16] Copas J, Dwan K, Kirkham JJ, Williamson PR (2014). A model-based correction for outcome reporting bias in meta-analysis. Biostatistics.

[CR17] Copas J, Marson A, Williamson PR, Kirkham JJ (2019). Model-based sensitivity analysis for outcome reporting bias in the meta analysis of benefit and harm outcomes. Statistical Methods in Medical Research.

[CR18] Coursol A, Wagner EE (1986). Effect of positive findings on submission and acceptance rates: A note on meta-analysis bias. Professional Psychology: Research and Practice.

[CR19] Dunn OJ, Clark V (1969). Correlation coefficients measured on the same individuals. Journal of the American Statistical Association.

[CR20] Dwan K, Altman DG, Arnaiz JA, Bloom J, Chan A-W, Cronin E, Siegfried N (2008). Systematic review of the empirical evidence of study publication bias and outcome reporting bias. PLOS ONE.

[CR21] Dwan K, Gamble C, Williamson PR, Kirkham JJ (2013). Systematic review of the empirical evidence of study publication bias and outcome reporting bias - an updated review. PLOS ONE.

[CR22] Eddelbuettel, D. (2013). Seamless R and C++ integration with RCPP. Springer. Retrieved from http://www.books24x7.com/marc.asp?bookid=69951

[CR23] Eddelbuettel D, Sanderson C (2014). RcppArmadillo: Accelerating R with high-performance C++ linear algebra. Computational Statistics and Data Analysis.

[CR24] Efron B, Tibshirani R (1993). An introduction to the bootstrap.

[CR25] Egger M, Smith GD, Schneider M, Minder C (1997). Bias in meta-analysis detected by a simple, graphical test. British Medical Journal.

[CR26] von Elm E, Röllin A, Blümle A, Huwiler K, Witschi M, Egger M (2008). Publication and non-publication of clinical trials: Longitudinal study of applications submitted to a research ethics committee. Swiss Medical Weekly.

[CR27] Elson M, Mohseni MR, Breuer J, Scharkow M, Quandt T (2014). Press CRTT to measure aggressive behavior: The unstandardized use of the competitive reaction time task in aggression research. Psychological Assessment.

[CR28] Fanelli, D. (2010). “Positive” results increase down the hierarchy of the sciences. *PLOS ONE,**5*, e10068. 10.1371/journal.pone.0010068 10.1371/journal.pone.0010068 PMC2850928 20383332

[CR29] Fanelli D (2012). Negative results are disappearing from most disciplines and countries. Scientometrics.

[CR30] Fernández-Castilla B, Declercq L, Jamshidi L, Beretvas SN, Onghena P, Van den Noortgate W (2021). Detecting selection bias in meta-analyses with multiple outcomes: A simulation study. The Journal of Experimental Education.

[CR31] Fisher RA (1921). On the “probable error” of a coefficient of correlation deduced from a small sample. Metron.

[CR32] Flake JK, Fried EI (2020). Measurement schmeasurement: Questionable measurement practices and how to avoid them. Advances in Methods and Practices in Psychological Science.

[CR33] Franco A, Simonovits G, Malhotra N (2016). Underreporting in psychology experiments: Evidence from a study registry. Social Psychological and Personality Science.

[CR34] Gerber S, Tallon D, Trelle S, Schneider M, Jüni P, Egger M (2007). Bibliographic study showed improving methodology of meta-analyses published in leading journals 1993–2002. Journal of Clinical Epidemiology.

[CR35] Ghersi, D. (2006). Issues in the design, conduct and reporting of clinical trials that impact on the quality of decision making. University of Sydney.

[CR36] Gleser LJ, Olkin I, Cooper H, Hedges LV, Valentine JC (2009). Stochastically dependent effect sizes. The Handbook of Research Synthesis and Meta-Analysis.

[CR37] Hahn S, Williamson PR, Hutton JL (2002). Investigation of within-study selective reporting in clinical research: Follow-up of applications submitted to a local research ethics committee. Journal of Evaluation in Clinical Practice.

[CR38] Hardwicke TE, Thibault RT, Kosie JE, Wallach JD, Kidwell MC, Ioannidis JPA (2022). Estimating the prevalence of transparency and reproducibility-related research practices in psychology (2014–2017). Perspectives on Psychological Science.

[CR39] Hedges LV, Olkin I (1985). Statistical methods for meta-analysis.

[CR40] Hedges LV, Tipton E, Johnson MC (2010). Robust variance estimation in meta-regression with dependent effect size estimates. Research Synthesis Methods.

[CR41] Hedges LV, Vevea JL, Rothstein HR, Sutton AJ, Borenstein M (2005). Selection method approaches. Publication bias in meta-analysis: Prevention, assessment, and adjustments.

[CR42] Higgins JPT, Thompson SG (2002). Quantifying heterogeneity in a meta-analysis. Statistics in Medicine.

[CR43] Higgins JPT, Thompson SG, Spiegelhalter DJ (2009). A re-evaluation of random-effects meta-analysis. Journal of the Royal Statistical Society.

[CR44] Higham NJ (2002). Computing the nearest correlation matrix-a problem from finance. IMA Journal of Numerical Analysis.

[CR45] Hilgard J, Engelhardt CR, Rouder JN, Segert IL, Bartholow BD (2019). Null effects of game violence, game difficulty, and 2D:4D digit ratio on aggressive behavior. Psychological Science.

[CR46] Hohn, R. E., Slaney, K. L., & Tafreshi, D. (2019). Primary study quality in psychological meta-analyses: An empirical assessment of recent practice. *Frontiers in Psychology,**9*,. 10.3389/fpsyg.2018.02667 10.3389/fpsyg.2018.02667 PMC6333691 30687152

[CR47] Houwelingen HCV, Arends LR, Stijnen T (2002). Advanced methods in meta-analysis: Multivariate approach and meta-regression. Statistics in Medicine.

[CR48] Hutton, J. L., & Williamson, P. R. (2000). Bias in meta-analysis due to outcome variable selection within studies. Journal of the Royal Statistical Society. Series C, 49, 359–370. Retrieved from http://www.jstor.org/stable/2680770

[CR49] Ishak KJ, Platt RW, Joseph L, Hanley JA (2008). Impact of approximating or ignoring within-study covariances in multivariate meta-analyses. Statistics in Medicine.

[CR50] Jackson D, Copas J, Sutton AJ (2005). Modelling reporting bias: The operative mortality rate for ruptured abdominal aortic aneurysm repair. Journal of the Royal Statistical Society: Series A.

[CR51] Jackson D, White IR (2018). When should meta-analysis avoid making hidden normality assumptions?. Biometrical Journal.

[CR52] Jackson D, White IR, Riley RD (2013). A matrix-based method of moments for fitting the multivariate random effects model for meta-analysis and meta-regression. Biometrical Journal.

[CR53] John LK, Loewenstein G, Prelec D (2012). Measuring the prevalence of questionable research practices with incentives for truth telling. Psychological Science.

[CR54] Lancee M, Lemmens CMC, Kahn RS, Vinkers CH, Luykx JJ (2017). Outcome reporting bias in randomized-controlled trials investigating antipsychotic drugs. Translational Psychiatry.

[CR55] LeBel EP, Borsboom D, Giner-Sorolla R, Hasselman F, Peters KR, Ratliff KA, Smith CT (2013). PsychDisclosure.org: Grassroots support for reforming reporting standards in psychology. Perspectives on Psychological Science.

[CR56] Moher, D., Liberati, A., Tetzlaff, J., Altman, D. G., & Group, T. P (2009). Preferred reporting items for systematic reviews and meta-analyses: The PRISMA statement. PLOS Medicine.

[CR57] O’Boyle EH, Gonzalez-Mule E, Banks GC (2017). The chrysalis effect: How ugly initial results metamorphosize into beautiful articles. Journal of Management.

[CR58] Open Science Collaboration. (2015). Estimating the reproducibility of psychological science. *Science,**349*. 10.1126/science.aac4716 10.1126/science.aac4716 26315443

[CR59] Przybylski AK, Weinstein N (2019). Violent video game engagement is not associated with adolescents’ aggressive behaviour: Evidence from a registered report. Royal Society Open Science.

[CR60] Rankin J, Ross A, Baker J, O’Brien M, Scheckel C, Vassar M (2017). Selective outcome reporting in obesity clinical trials: A cross-sectional review. Clinical Obesity.

[CR61] Raudenbush SW, Cooper H, Hedges LV, Valentine JC (2009). Analyzing effect sizes: Random-effects models. The Handbook of Research Synthesis and Meta-Analysis.

[CR62] Riley RD (2009). Multivariate meta-analysis: The effect of ignoring within-study correlation. Journal of the Royal Statistical Society: Series A (Statistics in Society).

[CR63] Rodgers MA, Pustejovsky JE (2021). Evaluating meta-analytic methods to detect selective reporting in the presence of dependent effect sizes. Psychological Methods.

[CR64] Schulze, R. (2004). Meta-analysis: A comparison of approaches. Hogrefe & Huber.

[CR65] Silberzahn R, Uhlmann EL (2015). Crowdsourced research: Many hands make tight work. Nature.

[CR66] Silberzahn R, Uhlmann EL, Martin DP, Anselmi P, Aust F, Awtrey E, Nosek BA (2018). Many analysts, one data set: Making transparent how variations in analytic choices affect results. Advances in Methods and Practices in Psychological Science.

[CR67] Simmons JP, Nelson LD, Simonsohn U (2011). False-positive psychology: Undisclosed flexibility in data collection and analysis allows presenting anything as significant. Psychological Science.

[CR68] Smyth RMD, Kirkham JJ, Jacoby A, Altman DG, Gamble C, Williamson PR (2011). Frequency and reasons for outcome reporting bias in clinical trials: Interviews with trialists. BMJ.

[CR69] Stanley TD, Doucouliagos H (2014). Meta-regression approximations to reduce publication selection bias. Research Synthesis Methods.

[CR70] Steegen S, Tuerlinckx F, Gelman A, Vanpaemel W (2016). Increasing transparency through a multiverse analysis. Perspectives on Psychological Science.

[CR71] Steiger, J. H. (1980a). Testing pattern hypotheses on correlation matrices: Alternative statistics and some empirical results. *Multivariate Behavioral Research,**15*, 335–352. 10.1207/s15327906mbr1503_7 10.1207/s15327906mbr1503_7 26794186

[CR72] Steiger, J. H. (1980b). Tests for comparing elements of a correlation matrix. *Psychological Bulletin,**87*, 245–251. 10.1037/0033-2909.87.2.245

[CR73] Sterling TD, Rosenbaum WL, Weinkam JJ (1995). Publication decisions revisited: The effect of the outcome of statistical tests on the decision to publish and vice versa. The American Statistician.

[CR74] Sterne JAC, Becker BJ, Egger M, Rothstein HR, Sutton AJ, Borenstein M (2005). The funnel plot. Publication bias in meta-analysis: Prevention, assessment and adjustments.

[CR75] Team, R. C. (2021). R: A language and environment for statistical computing. Retrieved from http://www.r-project.org/

[CR76] Tversky A, Kahneman D (1971). Belief in the law of small numbers. Psychological Bulletin.

[CR77] van Aert, R. C. M. (2022). Puniform: Meta-analysis methods correcting for publication bias. (0.2.5 ed.). Retrieved from https://cran.r-project.org/package=puniform

[CR78] van Aert, R. C. M., & van Assen, M. A. L. M. (2022). Correcting for publication bias in a meta-analysis with the p-uniform* method. Manuscript submitted for publication. 10.31222/osf.io/zqjr9 10.3758/s13423-025-02812-4 PMC12948804 41760983

[CR79] van Aert RCM, Wicherts JM, van Assen MALM (2016). Conducting meta-analyses on p-values: Reservations and recommendations for applying p-uniform and p-curve. Perspectives on Psychological Science.

[CR80] van Erp, S. J., Verhagen, J., Grasman, R. P. P. P., & Wagenmakers, E.-J. (2017). Estimates of between-study heterogeneity for 705 meta-analyses reported in psychological bulletin from 1990–2013. *Journal of Open Psychology Data,**5*. 10.5334/jopd.33

[CR81] Venables, W. N., & Ripley, B. D. (2002). Modern applied statistics with s (4th ed.). Springer.

[CR82] Viechtbauer, W. (2010). Conducting meta-analyses in r with the metafor package. *Journal of Statistical Software*, 36, 1–48. 10.18637/jss.v036.i03

[CR83] Wayant, C., Scheckel, C., Hicks, C., Nissen, T., Leduc, L., Som, M., & Vassar, M. (2017). Evidence of selective reporting bias in hematology journals: A systematic review. *PLOS ONE,**12*, e0178379. 10.1371/journal.pone.0178379 10.1371/journal.pone.0178379 PMC5453439 28570573

[CR84] Wicherts, J. M., Veldkamp, C. L. S., Augusteijn, H. E. M., Bakker, M., van Aert, R. C. M., & van Assen, M. A. L. M. (2016). Degrees of freedom in planning, running, analyzing, and reporting psychological studies: A checklist to avoid p-hacking. *Frontiers in Psychology,**7*. 10.3389/fpsyg.2016.01832 10.3389/fpsyg.2016.01832 PMC5122713 27933012

